# MiR-21-3p Promotes Hepatocellular Carcinoma Progression *via* SMAD7/YAP1 Regulation

**DOI:** 10.3389/fonc.2021.642030

**Published:** 2021-03-08

**Authors:** Yinghui Hong, Mingliang Ye, Fan Wang, Jun Fang, Chun Wang, Jie Luo, Jialiang Liu, Jing Liu, Lan Liu, Qiu Zhao, Ying Chang

**Affiliations:** ^1^ Department of Gastroenterology, Zhongnan Hospital of Wuhan University, Wuhan, China; ^2^ Clinical Center and Key Laboratory of Intestinal and Colorectal Diseases, Wuhan University, Wuhan, China

**Keywords:** MiR-21-3p, hepatocellular carcinoma, SMAD7, YAP1, prognosis

## Abstract

**Background:**

Hepatocellular carcinoma (HCC) remains a major global health burden due to its high prevalence and mortality. Emerging evidence reveals that microRNA (miRNA) plays a vital role in cancer pathogenesis and is widely involved in the regulation of signaling pathways *via* their targeting of downstream genes. MiR-21-3p, a liver-enriched miRNA, and SMAD7, the negative regulator of the TGF-*β* signaling pathway, likely exert a vital influence on HCC progression.

**Aims:**

Here, we explore the role of the miR-21-3p-SMAD7/YAP1 axis on HCC pathogenesis.

**Methods:**

MiRNA microarray analysis was performed for miRNA screening. The dual-luciferase assay was adopted for target verification. Expression of miRNA and related genes were quantified *via* qRT-PCR, western blotting, and immunohistochemical staining. Flow cytometry and the transwell migration assay were used to detail cell apoptosis, invasion and metastases. Rat models were established to explore the role of the miR-21-3p-SMAD7/YAP1 axis in hepatocarcinogenesis. Bioinformatics analysis was conducted for exploring genes of clinical significance.

**Results:**

MiR-21-3p levels were found to be significantly elevated in hepatocellular carcinoma and indicate poor overall survival. High miR-21-3p levels were associated with advanced tumor stages (*P* = 0.029), in particular T staging (*P* = 0.026). Low SMAD7/high YAP1 levels were confirmed in both HCC and rat models with advanced liver fibrosis and cirrhosis. Besides, SMAD7 was demonstrated to be the direct target of miR-21-3p. The effect of MiR-21-3p on tumor phenotypes and YAP1 upregulation could be partly reversed *via* the restoration of SMAD7 expression in HCC cell lines. Overexpression of YAP1 after miR-21-3p upregulation promoted expression of nuclear transcription effector connective tissue growth factor. Co-survival analysis indicated that lower miR-21-3p/higher SMAD7 (*P* = 0.0494) and lower miR-21-3p/lower YAP1 (*P* = 0.0379) group patients had better overall survival rates. Gene Set Variation Analysis revealed that gene sets related to miR-21-3p and SMAD7 were significantly associated with the TGF-β signaling pathway in HCC.

**Conclusion:**

MiR-21-3p promotes migration and invasion of HCC cells and upregulation of YAP1 expression *via* direct inhibition of SMAD7, underscoring a major epigenetic mechanism in the pathogenesis of HCC.

## Introduction

Hepatocellular carcinoma (HCC), with its rising global incidence, is a leading cause of cancer-related deaths and still lacks effective treatments (1). More than half of HCC patients are already in advanced stages of liver cancer on initial diagnosis (2). For patients suffering unresectable HCC, the multi-kinase inhibitors sorafenib and lenvatinib are considered to be effective drugs of treatment that target the multisystemic diseases (3). However, their rates of patient survival prolongation remain far from satisfactory (4). Metastasis is a crucial feature that distinguishes benign from malignant tumors, and migration and invasion of cancer cells remain the major mechanisms of drug resistance and tumor recurrence (5). It is thus of great importance to further investigate the mechanisms of HCC pathogenesis.

MicroRNAs (miRNAs), small endogenous non-coding ribonucleic acid molecules, are mainly involved in RNA silencing and regulation of gene expression *via* complementary targeting of the 3′-UTR region of mRNA (6). MiRNAs have been known to serve as both diagnostic and prognostic biomarkers for cancer since their discovery (7). Mutant p53 has previously been reported to facilitate the expression of miR-21-3p and further promote pulmonary metastasis (8). In addition, miR-21-3p has been found to play a pivotal role in maintaining cancer cell stemness in the progression of esophageal squamous cell carcinoma (9). We previously reported that the results of miRNA microarray analysis revealed miR-21-3p to be significantly upregulated in HCC. Moreover, prior studies reported miR-21-3p to influence HCC growth *via* direct downregulation of adenosyltransferases 2A and 2B (MAT2A/MAT2B) (10). However, other key targets of miR-21-3p await elucidation.

The SMAD7 protein is the negative regulator of the transforming growth factor *β* (TGF-*β*) signaling transduction pathway; the protein exerts its action *via* binding TGF-*β* Receptor I (T*β*RI) and interfering with recruitment of the SMAD2/SMAD3 (R-SMAD) complex and blocking functional SMAD complexes from interacting with intranuclear DNA (11, 12). A “double-edged sword” involved in maintaining physiologic cell proliferation and differentiation, the TGF-*β* signaling pathway is also key in tumor pathogenesis (13). Dysfunction of SMAD7 and excessive activation of the TGF-*β* signaling pathway were reported to be common in various types of cancers (14, 15). Feng et al.’s team found that SMAD7 deletion accelerates HCC tumorigenesis in mouse models *via* activation of the STAT3 signaling pathway (16). The loss of function of SMAD7 was also reported to promote HCC cell proliferation, accelerate the G1-S phase transition and reduce cell apoptosis *in vivo* apoptosis (17).

Yes-associated protein 1 (YAP1) is the “star nuclear effector” of the Hippo pathway, which consists of a series of kinases (18). YAP1 is in hyperactive status and considered to be an oncogene in several types of solid tumors, including HCC (19). SIX4 promotes hepatocellular carcinoma metastasis through upregulating YAP1 (20). LncRNA B4GALT1-AS1 facilitates colon cancer cell stemness *via* recruiting YAP1 to the nucleus and enhancing YAP1 transcriptional activity (21). Cytoplasmic YAP1 was previously reported to partially inhibit TGF-*β* signal transduction in COS-7 cells (a CV-1 African green monkey fibroblast cell line transformed with a mutant strain of Simian Virus 40 (SV40)) *via* enhancement of SMAD7 binding to T*β*RI. Besides, YAP1 was demonstrated to be a novel SMAD7-interacting protein (22). Nevertheless, the relationship between SMAD7 and YAP1 in HCC pathogenesis remains unclear.

Here, we detail the consequences of miR-21-3p overexpression in human HCC cells. We also describe the facilitation of YAP1 expression and the subsequent promotion of malignant phenotype progression by miR-21-3p *via* direct targeting of SMAD7.

## Materials and Methods

### Tissue Collection and Cell Culture

16 pairs of HCC tissue samples and background liver tissue (BL) were obtained from patients suffering HCC who underwent partial hepatectomy between January 2019 and December 2020. After surgical resection, specimens were immediately snap-frozen in liquid nitrogen. RNA-later (#AM7201, ThermoFisher, USA) was added to the cryogenic vials to prevent RNA degradation for subsequent extraction. Collection and usage of these samples followed ethical and institutional guidelines; all protocols were implemented after obtaining written informed consent from donors. This study was approved by the local Zhongnan Hospital Ethics Committee of Wuhan University (Approval No.2018078).

Cell lines (L02, Huh7, HepG2, HCCL-M3) involved in this experiment were purchased from Stem Cell Bank, Chinese Academy of Sciences. A mixture of Dulbecco’s Modified Eagle’s Medium (ThermoFisher), 10% fetal bovine serum (ThermoFisher), and 1% penicillin–streptomycin were used in cell culturing. All cells were grown in a humidified environment at 5% CO_2_ and 37°C.

### MiRNA Microarray Analysis

Microarray data adopted for analysis have been described in detail before (23). Data were uploaded to NCBI’s Gene Expression Omnibus (GEO) public database http://www.ncbi.nlm.nih.gov/geo/, accession number, GSE20077).

### RNA Extraction, Reverse Transcription and qRT-PCR

Total RNA extraction was performed using Trizol Reagent (Invitrogen, USA) according to manufacturer instructions. Detection of RNA quality and concentration was accomplished using the Nanodrop 2000 spectrophotometer (ThermoFisher); cDNA was obtained *via* a reverse transcription reaction with relevant kits (Toyobo, Japan). Gene and miRNA expression were examined using UltraSYBR Mixture (CWbio, China) and the CFX96Touch RT-PCR system (Biorad, USA). The mRNA levels of miR-21-3p and relevant genes were normalized to U6 (Ribobio, China) and GAPDH, respectively. The relative expression ratio was calculated using the 2^−ΔΔCq^ method. Primers used are listed in **Table S1**.

### MiRNA Related Reagents, Plasmid Construction, and Cell Transfection

Gain and loss of function of miR-21-3p were accomplished by transfecting miRNA mimics and inhibitors, respectively. Mimic- (mimic-NC) and inhibitor- (in-NC) negative controls were purchased from Ribobio. The complementary strand of miR-21-3p served as an inhibitor of miR-21-3p and competed for binding sites between miR-21-3p and target genes, but would not decrease the expression levels of miRNAs. The final concentration of these reagents was 50 nM. The SMAD7 expression vector (P-SMAD7) was constructed using a GV141 vector (GeneChem, China) with plasmids-NC (P-NC) serving as an experimental control. All transfection experiments were performed using Lipofectamine 2000 (Invitrogen, USA) according to manufacturer guidelines.

### Western Blot

Proteins were collected using lysis buffer (Beyotime Biotechnology, China) containing protease inhibitor (PMSF, 1:100) and were centrifuged at 20627g for 20 min at 4°C; the supernatant was subsequently transferred to new tubes. Protein concentrations were detected using a BCA protein kit (Beyotime). Lysis buffer was mixed with protein loading buffer and protein was denatured at 100°C for 5 min. An equal amount of proteins was separated using SDS-polyacrylamide gels and then transferred to polyvinylidene fluoride (PVDF) membranes (Millipore, USA). Membranes were blocked for 2 h with 5% skimmed milk and incubated with primary antibodies overnight at 4°C. Secondary antibodies were subsequently added for 1.5 h at room temperature. Protein bands were obtained using an enhanced chemiluminescence (ECL) kit (Servicebio, Wuhan, China) using GENESys (Synoptics Ltd, China). anti-SMAD7 (#25840-1-AP) was purchased from Proteintech (China). anti-YAP1 (#14074), anti-E-cad (#3195), anti-N-cad (#13116), anti-vimentin (#5741) and anti-CTGF (#86641); these antibodies were purchased from Cell Signaling Technologies (USA). Anti-Bcl2 (#ab182858), anti-Bax (#ab32503) and anti-GAPDH (#ab8245) antibodies were purchased from Abcam (UK) while Goat Anti-Rabbit IgG HCS (#A25222) and goat anti-mouse IgG-horseradish peroxidase (#A25012) were purchased from Abbkine (USA).

### Dual-Luciferase Reporter Assay

A pmirGLO-SMAD7 vector was constructed, consisting of predicted binding sites that were mutated and ligated between the PmeI and XbaI restriction enzymes sites of the pmirGLO Dual-Luciferase miRNA Target Reporter Vector (#E1300, Promega, USA). The SMAD7 3′-UTR region contains two putative binding sites for miR-21-3p, with seed regions at 1,428–1,434 and 1,445–1,451. The mutant 3 (Mut 3) strand mutated two binding sites simultaneously. The wild-type (WT) and mutant SMAD7 3′-UTR luciferase reporter plasmids (0.25 μg/well) were co-transfected into huh-7 cells with miR-21-3p mimics or miR-NC (50 nM, 0.15 μl/well), respectively. A Dual-Glo Luciferase Assay System (#E2920, Promega) was employed to investigate firefly luciferase and Renilla luciferase activity 24 h after transfection, respectively. Firefly luciferase activity was normalized to Renilla luciferase activity. Data were collected using Enspire 2300 (PerkinElmer). Six repetitions per group were calculated.

### Cell Apoptosis and Flow Cytometry Assays

A total of 4 × 10^5^ cells were transfected with miR-21-3p mimics with/or SMAD7 plasmids. After 24 h, cells were collected (including dead cells in culture medium); Huh-7 and Hep-G2 cells were processed according to Annexin V-FITC/PI Double stain apoptosis detection kit manufacturer instructions (#4101-2, BestBio, China). Fluorescence intensity on the flow cytometer was promptly collected to investigate the rate of apoptosis (Cytoflex Beckman, China).

### Cell Invasion and Metastasis Assay

The protocol for processing HCC cell lines was identical to the above cell apoptosis assay. Cells were harvested at 24 h post-transfection, suspended in 5% FBS culture medium and seeded at 5 × 10^4^ cells per upper chamber (6.5 mm. in diameter, 8.0 um pore size, Corning, USA). The lower chamber was filled with 600 μl of medium (15% FBS). Cells were fixed with 4% paraformaldehyde for 20 min at room temperature and stained with 0.1% crystal violet (Sigma-Aldrich, USA). The upper cells were wiped with cotton swabs. Random photographs of 200× fields were taken using an inverted microscope (Olympus IX3). The difference between cell invasion and migration assays was that in the latter, one upper chamber required pretreatment with 0.3% Matrigel matrix (#356234, Corning); this was performed at 37°C for 4 h. Three independent experiments were performed.

### Animal Experiments

A Wistar rat model was established as previously described and rats were grouped according to the Metavir score system (24, 25). In brief, Wistar rats (male, aged 7–8 weeks and weighing 200–220 g) were purchased from Beijing Vital River Laboratory Animal Technology Co., Ltd (China). All animal handling methods and experimental procedures were approved by the Animal Care and Use Committee of Wuhan University in accordance with the Animal Experiment/Animal Biosafety Level-III Laboratory Guidelines. Rats were injected with 40% carbon tetrachloride (CCl4) dissolved in maize oil (1.5ml/kg) twice per week. At 8, 14, and 18 weeks, liver fibrosis, cirrhosis, and HCC manifested, respectively. Hematoxylin–eosin and Masson staining images of rat tissues are shown in **Figure S1A**.

### Immunohistochemistry

Immunohistochemical staining of patient and animal tissues was performed by the Servicebio Company. Tissue photographs were taken using an inverted microscope (Olympus IX3). Positively stained cells were counted in at least five fields from each area at 400× magnification. Primary antibodies were:

Anti-SMAD7: (#25840-1-AP, Proteintech): 1:200; anti-YAP1 (#14074, Cell Signaling Technology): 1:400.

### Gene Set Variation Analysis and Kyoto Encyclopedia of Genes and Genomes Pathways Analysis With miR-21-3p/SMAD7 Expression

GSVA, an open-source software package for R (3.5.2), was adopted to explore the potential biological relevance of genes according to their levels of expression. Findings are presented in the form of the intuitive volcano and specific heat maps. Gene terms with |logFC| ≥0.1 and *P <*0.05 were considered statistically significant. The KEGG gene sets (c2.cp.kegg.v6.2.symbols.gmt) downloaded from the Molecular Signatures Database–MsigDB (http://www.broad.mit.edu/gsea/msigdb/index.jsp) were used for miRNA enrichment analysis.

### Bioinformatics Data

MicroRNA Target Prediction Database (miRDB), PicTar and TargetScan databases were used for searching miRNA targets and predicting binding sites. Cohort data, including RNA sequencing and clinical data of 376 HCC patients were downloaded from The Cancer Genome Atlas (TCGA) database (https://portal.gdc.cancer.gov/). Data with incomplete clinical information were excluded during analysis. Clinical characteristics of HCC patients from the TCGA database are detailed in **Table 1**.

**Table 1:** Clinical characteristics of HCC patients from the studied TCGA data set.

Characteristic	Group	Case	Percentage (%)	Total (N)
Age	<60 ≥60	172 204	45.70% 54.30%	376
Gender	Male Female	254 122	67.60% 32.40%	376
Status	Live Dead	248 128	66.00% 34.00%	376
Grade	G1 + G2 G3 + G4 Unknown	235 136 5	62.50% 36.20% 1.30%	376
Stage	I II III+IV Unknown	175 86 91 24	46.50% 22.90% 24.20% 6.40%	376
T	T1 T2 T3 T4 Unknown	185 94 81 13 3	49.20% 25.00% 21.50% 3.50% 0.80%	376
N	N0 N1 NX	257 4 115	68.40% 1.00% 30.60%	376
M	M0 M1 MX	272 4 100	72.30% 1.10% 26.60%	376

### Statistical Analysis

Data were presented as mean ± standard deviation (SD) and analyzed using the Student’s t-test or one-way ANOVA. Quantitative data were representative of three experiments. Wilcoxon signed-rank and Wilcoxon rank-sum tests were adopted to analyze gene expression in paired and non-paired tissue samples. The relationship between clinicopathological characteristics and gene expression was evaluated using the Wilcoxon rank-sum test. Prognosis analysis was performed using the Kaplan–Meier method and univariate Cox regression. All statistical analyses and data plotting were performed using R (v.3.5.2); *P*< 0.05 was considered statistically significant.

## Results

### The Upregulation of miR-21-3p in HCC and Its Clinical Significance Based on Bioinformatics Analysis

Based on our earlier chip analysis results, which were sorted by *P*-value and differential expression, miR-21-3p was found to be significantly (*P* = 0.0278) upregulated in HCC as compared to normal liver tissue (**Table 2**). To further verify chip analysis results, miR-21-3p mRNA expression was examined in both human HCC as well as Wistar rat model tissues, respectively. Results revealed miR-21-3p to have been significantly enriched in human HCC tissues as compared to background liver tissues [**Figure 1A(a)**]. Such upregulation was also observed in tissues obtained from rats with late-stage liver fibrosis and cirrhosis [**Figure 1A(b)**]. Relative to normal human L02 cells, miR-21-3p levels were increased in Huh-7, Hep-G2, and HCCL-M3 HCC cell lines [**Figure 1A(c)**]. Considering expression consistency in human tissues, rat liver disease models and cell lines, miR-21-3p upregulation was further investigated. Data downloaded from TCGA, including the clinicopathological characteristics of 376 HCC patients (**Table 1**), were divided into high and low miR-21-3p expression groups. Patients with high miR-21-3p expression were significantly correlated with advanced clinical stages (*P* = 0.029), especially higher T staging (*P* = 0.026), while no significant differences were observed in patient age or gender (**Figure 1B**). Higher miR-21-3p expression was also correlated to shorter 10-year overall survival (OS) time (log-rank *P* = 0.026) (**Figure 1C**). GSVA analysis of miR-21-3p displayed that high miR-21-3p expression was mainly enriched among 20 pathways in the progression of HCC (**Figure 1D**). The top three relevant gene sets were involved in nitrogen metabolism, fatty acid metabolism and primary bile acid biosynthesis, underscoring the vital influence miR-21-3p exerts on cellular metabolic activity (**Figure 1E**). KEGG pathway analysis of miR-21-3p targets revealed that downstream targets were strongly associated with metabolic pathways, the Hippo signaling pathway and TGF-β transduction pathway (**Table 3**). The intersections of miR-21-3p potential targets from three authoritative databases, namely TargetScan, PicTar, and miRDB, were SMAD7, HBP1, and FBXO11, respectively (**Figure 1F**). Of these, SMAD7 scored highest (**Table 4**). The relationship between miR-21-3p and SMAD7, however, requires further elucidation.

**Table 2:** Levels of miRNA expression in all three HCC cases and corresponding normal liver samples (top 10 listed here).

No.	#Term	diff_(HCC^a^ *vs* NLT^b^)	P-value	Correct_P
1	hsa-miR-34a	2.8754	0.0023	0.0189
2	hsa-miR-224	5.6242	0.0025	0.0189
3	hsa-miR-34b	2.5661	0.0050	0.0252
4	hsa-miR-21-3p	1.9786	0.0059	0.0278
5	hsa-miR-222	2.5337	0.0316	0.0957
6	hsa-miR-513a-5p	2.0848	0.0508	0.0300
7	hsa-miR-500a-3p	1.8265	0.0758	0.1278
8	hsa-505-5p	1.7973	0.1086	0.1496
9	hsa-miR-18a	0.1896	0.1896	0.2054
10	hsa-miR-500	1.7753	0.1898	0.2054

^a^hepatocellular carcinoma; ^b^normal liver tissue.

**Figure:**
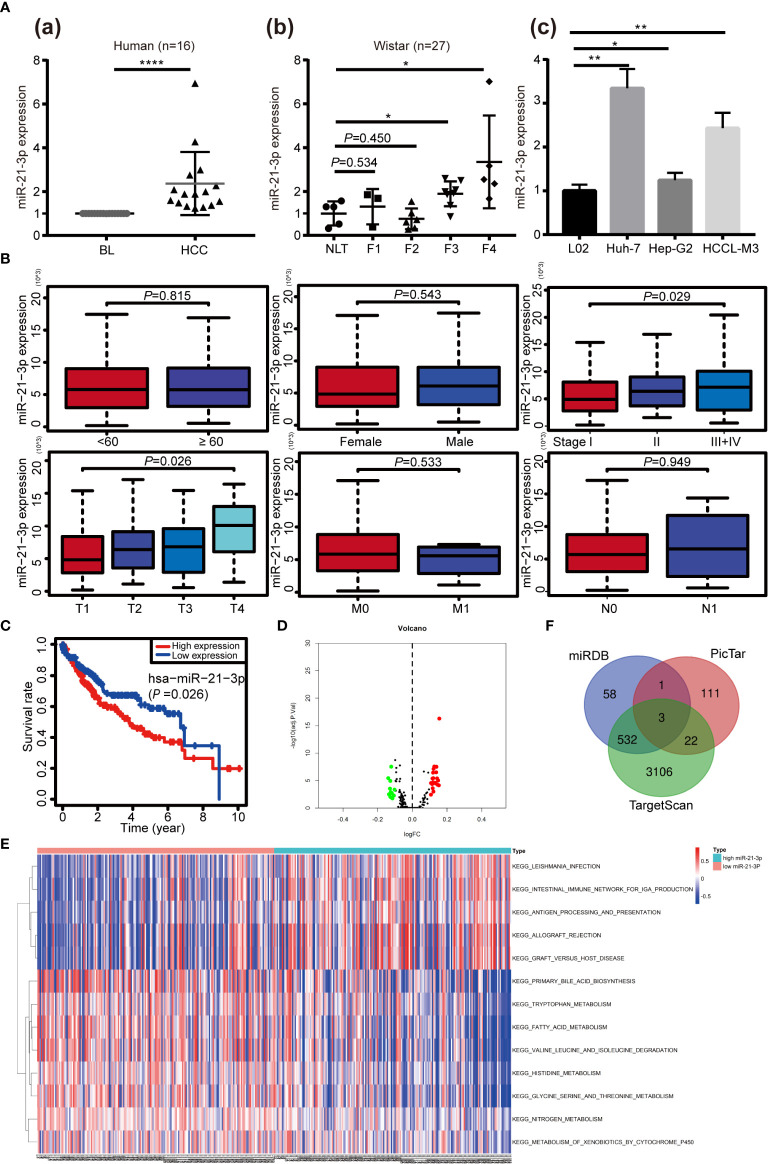
**Figure 1** Upregulation and clinical significance of miR-21-3p in hepatocellular carcinoma (HCC). **(A)** TaqMan quantitative RT-PCR was performed to analyze microRNA-21-3p (miR-21-3p) expression; 16 pairs of human HCC and counterpart background liver (BL) tissue were examined **(A**-a**)**; Normal liver tissue (NLT), fibrotic (F1–F3) and cirrhotic (F4) tissue from rat models were also analyzed **(A**-b**)**; MiR-21-3p expression in L02, Huh-7, Hep-G2 and HCCL-M3 cell lines **(A**-c**)**; Each dot indicates the level of expression in an individual case, calculated by the 2^−ΔΔCt^ method. **(B)** Association of the miR-21-3p expression level with clinicopathological characteristics (age, gender, and TNM staging, respectively). **(C)** Kaplan–Meier curves representing the relationship between miR-21-3p and the overall survival (OS, as percentage) in HCC patients among the studied data set from The Cancer Genome Atlas (TCGA) (n = 376). Statistical significance between miRNA expression and OS was determined using the log-rank test. **(D)** Volcano map of the microarray gene set variation analysis (GSVA) data based on high or low miR-21-3p expression. **(E)** Kyoto Encyclopedia of Genes and Genomes pathways of miR-21-3p, based on TCGA data GSVA in HCC. Significant terms are described in the heat map (top 13 listed). **(F)** Venn diagram of the intersections of three databases (miRDB, TargetScan, and PicTar) of predicted downstream target genes of miR-21-3p. *P < 0.05, **P < 0.01, ****P < 0.0001.

**Table 3:** The Kyoto Encyclopedia of Genes and Genomes (KEGG) pathway enrichment analysis of miR-21-3p downstream targets (top 12 listed here).

#Term	ID	Input number	Background number	P-Value	Corrected P-Value
Metabolic pathways	hsa01100	28	1243	0.0068	0.0303
Pathways in cancer	hsa05200	14	397	0.001	0.0110
MAPK signaling pathway	hsa04010	12	255	0.0003	0.0063
PI3K-Akt signaling pathway	hsa04151	12	342	0.0030	0.0173
cGMP-PKG signaling pathway	hsa04022	11	167	2.89E-05	0.0016
Ubiquitin mediated proteolysis	hsa04120	10	137	2.93E-05	0.0016
Insulin signaling pathway	hsa04910	10	139	3.29E-05	0.0016
Hippo signaling pathway	hsa04390	10	154	7.42E-05	0.0022
cAMP signaling pathway	hsa04024	10	199	0.0005	0.0085
HTLV-I infection	hsa05166	10	259	0.0034	0.01838
TGF-beta signaling pathway	hsa04350	6	84	0.0013	0.0110
mTOR signaling pathway	hsa04150	7	154	0.0060	0.0269

**Table 4:** Intersection of potential miR-21-3p targets based on analysis of three online databases.

Target Gene	TargetScan total context score	miRDBtarget score	PicTar score
SMAD7	−0.39	92	2.15
HBP1	−0.15	53	2.22
FBXO11	−0.23	58	2.79

### SMAD7, a Direct Target of miR-21-3p, Was Decreased in HCC

**Figure 2:**
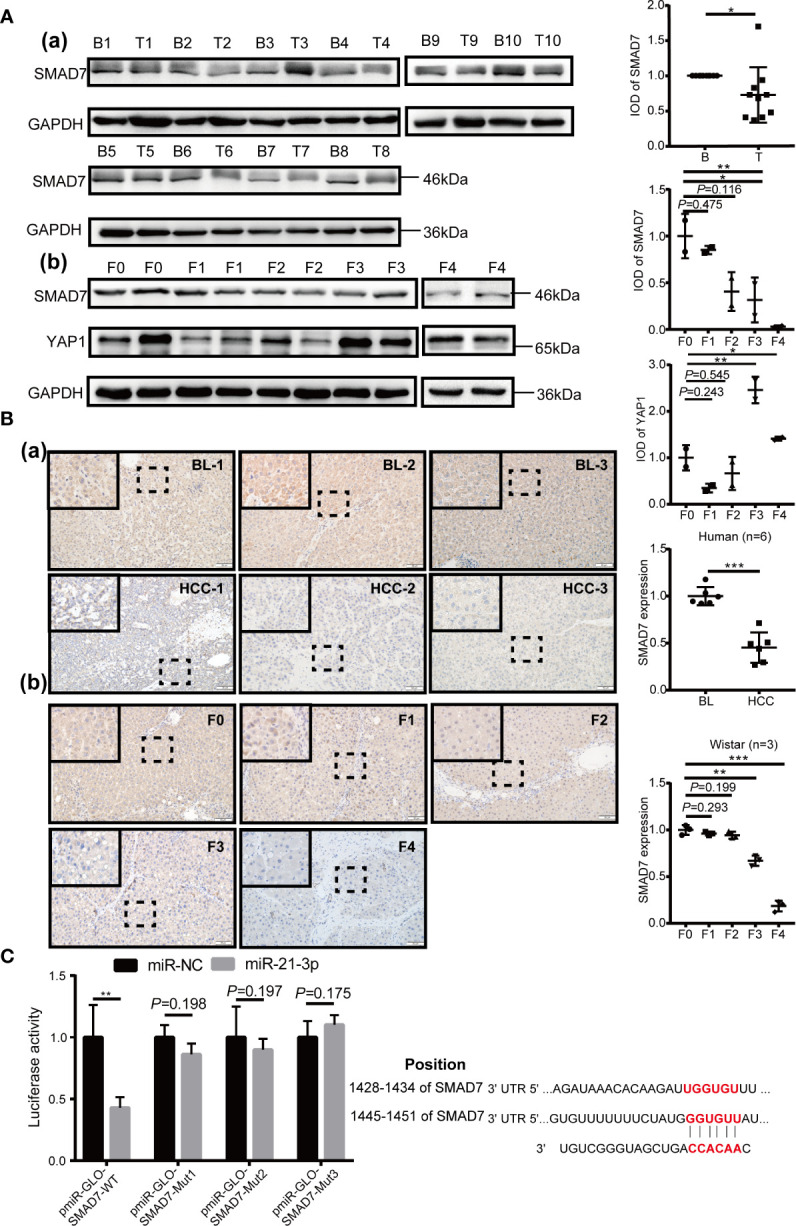
SMAD7, a direct target of miR-21-3p, was decreased in HCC. **(A)** Western blotting and density photography of SMAD7 expression in human HCC (T: tumor) and background liver tissue (B: background) (a); SMAD7 and YAP1 expression in different stages of rat hepatocarcinogenesis, including normal, fibrotic (F1–F3) and cirrhotic (F4) tissue (b). **(B)** SMAD7 expression in HCC (a) and rat liver tissue in different stages of tumorigenesis (b) as illustrated by immunohistochemical methods (magnification 400×). Six paired human and three rat samples from each group were statistically analyzed. **(C)** Base pairing complement detailed the two putative YAP1 miR-21-3p binding sites at 3’-UTR predicted by TargetScan. PmiR-GLO-SMAD7 vector was co-transfected with mimic-21 or mimic-NC; wild-type (WT); or mutant (Mut). Mut-1-2 represents the two predicted binding sites. Mut-3 represents two mutant binding sites. Luciferase activity assay of pmiR-GLO-SMAD7-3’-UTR co-transfection with miR-21-3p or NC mimics. Firefly luciferase activity was measured 24 h after transfection and normalized to Renilla luciferase activity. All data for miR-NC groups were set as 1. All experiments were performed in triplicate. Data are presented as mean ± SEM. *P < 0.05,**P < 0.01, ***P < 0.001.

The KEGG enrichment results of miR-21-3p and prediction of potential target scores were considered when evaluating expression of SMAD7 in human and rat model tissue samples, respectively. Compared to background liver tissue, SMAD7 was decreased in HCC (9/10) [**Figure 2A(a)** **]**. In addition, lighter brown staining was observed in HCC as compared to background liver tissue [**Figure 2B(a)**]. Along with the progression of liver disease in rat models, SMAD7 deletion in rats with advanced fibrosis and cirrhosis (F3, F4) (F: fibrosis) was observed in terms of protein expression levels when compared to normal rats [**Figure 2A(b)**]; immunohistochemical staining confirmed this trend [**Figure 2B(b)**]. Considering the apparent up-regulation of miR-21-3p and down-regulation of SMAD7 in HCC, the Dual-Luciferase assay was performed in Huh-7 cells to confirm the linear regulatory association among miR-21-3p and SMAD7. Binding site prediction revealed two high-scoring potential binding sites in the 3′-UTR region of SMAD7. In the miR-21-3p and reporter vector containing wild-type SMAD7 co-transfection group, luciferase activity was found to be significantly inhibited, while in the group containing mutant SMAD7 reporter vectors, the inhibition efficiency was not found to be statistically significant between miR-NC and miR-21-3p groups (**Figure 2C**). The above findings indicate that the predicted binding sites likely both inhibit SMAD7.

### Overexpression of SMAD7 Partly Abrogates the Tumorigenic Effect of miR-21-3p on Malignant Phenotypes in HCC

**Figure 3:**
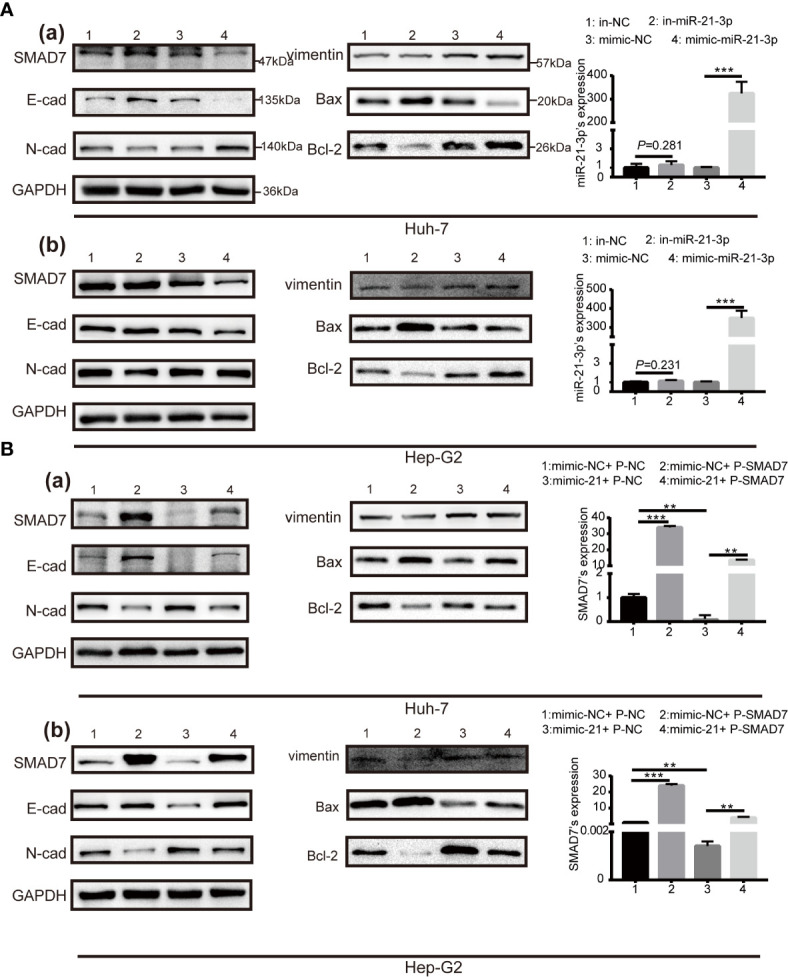
Cell phenotype biomarker variation after SMAD7 transfection with or without miR-21-3p mimics. **(A)** Western blotting revealing expression of the pro-apoptotic proteins Bax and Bcl-2 and the epithelial–mesenchymal transition (EMT) proteins E-cadherin, N-cadherin and vimentin 48 h after transfection with miR-21-3p inhibitors with or without mimics in Huh-7 (a) and Hep-G2 (b) cells, respectively. Transfection efficiency is listed alongside the image. **(B)** SMAD7 plasmids (P-SMAD7) were constructed to overexpress SMAD7; plasmids-NC (P-NC) served as control. Transfection efficiency of SMAD7 is listed alongside the image. Pro-apoptotic (Bcl-2, Bax) and EMT (E-cadherin, N-cadherin, vimentin) biomarkers were quantified after P-SMAD7 and/or miR-21-3p mimic co-transfection in Huh-7 (a) and Hep-G2 (b) cells; photo density in group 1 (mimic-NC+ P-NC) was set at 1. All experiments were performed in triplicate. Data are presented as mean ± SEM. **P < 0.01, ***P < 0.001.

**Figure 4:**
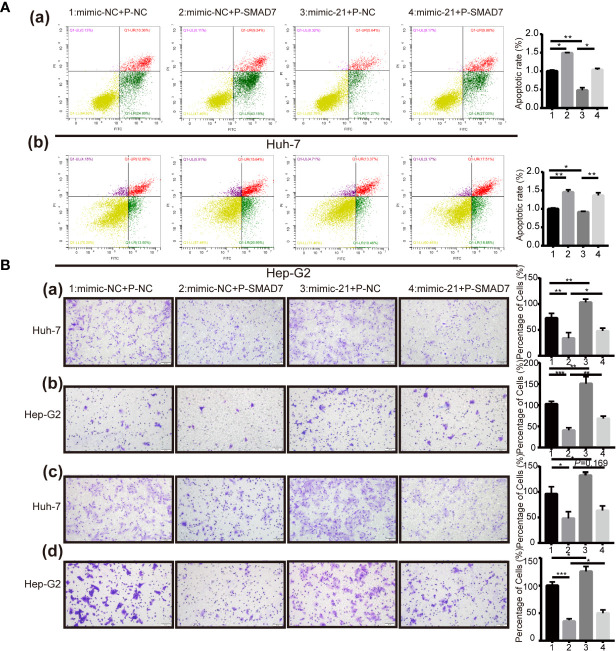
Effects of miR-21-3p on malignant HCC phenotypes could be partly reversed *via* SMAD7 overexpression. **(A)** Flow cytometry was performed to analyze the apoptotic index of Huh-7 (a) and Hep-G2 (b) cells after transfection. Cells were co-stained with Annexin FITC/PI; Annexin FITC+/PI- cells that stained green were considered as being in early apoptosis. Normal, late-stage apoptotic and necrotic cells are shown in yellow, red and purple, respectively. Three independent experiments were performed. Data are shown as mean ± SD. **(B)** Transwell assay was performed to detect the migration (a, b) and invasion (c, d) in Huh-7 and Hep-G2 cells separately after transfection. *P < 0.05, **P < 0.01; ***P < 0.001.

Expression of SMAD7 increased after transfection of miR-21-3p inhibitors and decreased following transfection of miR-21-3p mimics in Huh-7 [**Figure 3A(a)**] and Hep-G2 [**Figure 3A(b)**] cells. To investigate the effects of the miR-21-3p/SMAD7 axis on HCC malignant phenotypes, biomarkers associated with cell apoptosis, migration, and invasion were studied. The pro-apoptotic protein Bax and epithelial signature protein E-cadherin (E-cad) were found to be upregulated, while the anti-apoptotic protein Bcl-2, mesenchymal characteristic protein N-cadherin (N-cad) and vimentin were downregulated after down-regulating miR-21-3p in Huh-7 and Hep-G2 cells, respectively (**Figure 3A**). Effects of miR-21-3p on malignant cellular biomarkers were partly attenuated by SMAD7 co-transfection in both cell lines (**Figure 3B**). Flow cytometry results revealed that rates of early apoptosis, as evidenced by green staining, were increased after SMAD7 transfection (*P* < 0.01) (**Figure 4A**). Migration [**Figure 4B (a-b)**] and invasion [**Figure 4B(c-d)**] were enhanced by transfection of miR-21-3p mimics and partly reversed by SMAD7 restoration.

### MiR-21-3p Enhanced YAP1 Expression Partly *via* Downregulation of SMAD7

**Figure 5:**
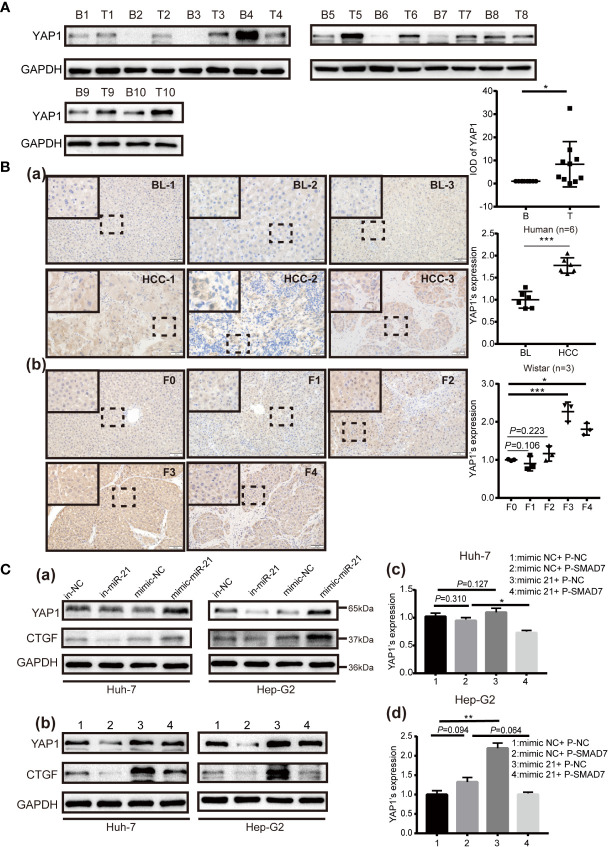
YAP1 is upregulated in HCC and is facilitated by miR-21-3p *via* SMAD7 inhibition. **(A)** Western blotting was performed to show YAP1 expression in human HCC (T: tumor) and corresponding background liver tissue (BL) (B: background). Photographs are detailed alongside. **(B)** YAP1 expression in HCC, BL (a) and different liver disease stages in a rat model (b) was evaluated *via* immunohistochemical staining (magnification 400×). Six paired human and three rat samples in each group were statistically analyzed. **(C)** Western blotting of YAP1 48 h after transfection with miR-21-3p inhibitors or mimics in Huh-7 and Hep-G2 cells (a); protein (b) and mRNA (c, d) YAP1 expression after plasmid-SMAD7 transfection, with or without miR-21-3p mimics. Experiments were performed separately in triplicate. *P < 0.05, **P < 0.01; ***P < 0.001.

KEGG pathway enrichment analysis revealed that miR-21-3p associates significantly with the Hippo/YAP1 pathway (**Table 3**). Notably, a prior study reported cytoplasmic YAP1 to enhance SMAD7 binding to activated T*β*RI (22). To further investigate potential associations among, miR-21-3p, SMAD7 and YAP1 in HCC, YAP1 expression was detected in both human and rat model HCC tissues. Compared to background liver tissue, YAP1 was found to be enriched in human HCC tissues (8/10) (**Figure 5A**), as well as in advanced liver fibrosis and cirrhosis tissues compared to early stages (F1, F2) (F: fibrosis) [**Figure 2A(b)**]. Darker brown staining on immunohistochemistry was observed in HCC tissues [**Figure 5B (a)**] as well as F3 and F4 stages in rat tissues as compared to F1 [**Figure 5B (b)**]. In both Huh-7 and Hep-G2 cells, YAP1 expression was upregulated by miR-21-3p and inhibited after reducing miR-21-3p [**Figures 5C(a, b)**]. On the protein level, promotion of YAP1 expression was partly attenuated by co-transfecting SMAD7 [**Figure 5C(b)**]. However, neither miR-21-3p nor SMAD7 had any significant influence on YAP1 mRNA levels [**Figures 5C (c, d)**]. Besides, mRNA (**Figure S2A**) and protein [**Figure 5C(b)**] expression trends of connective tissue growth factor (CTGF), previously demonstrated to be the direct nuclear target of YAP1, were consistent with YAP1 here (26).

### The Clinical Significance of SMAD7/YAP1 Based on Bioinformatics Analysis

**Figure 6:**
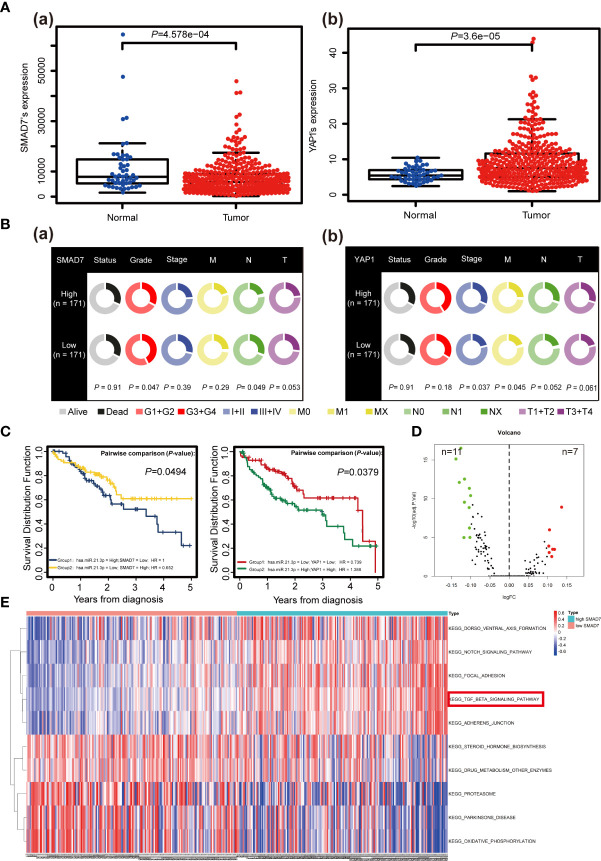
The clinical significance of SMAD7/YAP1 in HCC based on bioinformatics analysis. **(A)** Discrepancies in SMAD7 (a) and YAP1 (b) expression levels in liver tumor tissues (Tumor) and non-paired relative normal samples (Normal) from the TCGA database are presented. **(B)** Relationship between expression of SMAD7 (a), YAP1 (b) and HCC clinicopathological terms as quantified using the Wilcoxon rank-sum test. Data with incomplete clinical traits were excluded from analysis. **(C)** Kaplan-Meier curves representing overall survival (OS, as percentage) in HCC patients based on miR-21-3p/SMAD7 or miR-21-3p/YAP1 levels from the studied TCGA data set (n = 376). Statistical significance among miRNA/mRNA expression and OS was determined using the log-rank test. SMAD7-related KEGG pathways based on gene set variation analysis of TCGA data are shown in volcano **(D)** and heat **(E)** maps (top ten including representative terms), respectively.

SMAD7 was decreased (*P* = 4.578e-04) and YAP1 (*P* = 3.6e-05) was increased in HCC compared to adjacent normal liver tissues based on data of 376 HCC patients downloaded from TCGA database (**Figure 6A**). Analysis of RNA sequencing and patient clinicopathological data revealed that higher levels of SMAD7 associate with lower grades (*P* = 0.047) [**Figure 6B (a)**]. In addition, higher YAP1 levels were found to associate with higher disease stages (*P* = 0.037), in particular, M stages (*P* = 0.045) [**Figure 6B(b)**]. Co-survival analysis revealed that lower miR-21-3p/higher SMAD7 (*P* = 0.0494) and lower miR-21-3p/lower YAP1 (*P* = 0.0379) ratios associate with a better five-year OS rate (**Figure 6C**). GSVA data, presented as a volcano map, revealed SMAD7 to be mainly involved in 18 pathways (**Figure 6D**). Among them, the TGF-*β* signaling pathway and the Notch signaling pathway were most relevant in HCC (**Figure 6E**).

## Discussion

As the second leading cause of cancer-related death worldwide, HCC still lacks efficient treatments. Pathological miRNA expression plays a critical role in cancer pathogenesis. According to data obtained from our microarray analysis, miR-21-3p ranked fourth based variance and *P*-values, while miR-34a, miR-224 and miR-34b ranked top three, respectively. The miR-34 family was reported to associate with DNA methylation in HCC (27), while miR-224 was reported to promote HCC migration and invasion *via* Homeobox D gene targeting (28). Prior literature primarily focused on introducing the function of the guide strand of miR-21, miR-21-5p, in HCC; not much data concerning the passenger strand miR-21-3p were detailed. Increasing studies have detailed the function of miR-21-3p, thereby furthering knowledge of the role this miRNA plays in cellular signaling (29, 30). Importantly, miR-21-3p may be used as a signature in predicting the survival rate of triple-negative breast cancer patients (31). In addition, its overexpression facilitates pulmonary metastasis by compromising the junction between tumor and stromal cells (8). Here, miR-21-3p levels were found to be increased in HCC tissue samples compared to adjacent background liver tissue (*P* < 0.001), suggesting a poor prognosis. Furthermore, mRNA expression level analysis of miR-21-3p in rat models revealed that miR-21-3p is involved in not only HCC progression but also tumorigenesis. KEGG pathway analysis of miR-21-3p binding targets revealed a significant association with the TGF-*β* transduction and Hippo signaling pathways. The abnormal overexpression of miR-21-3p perpetuated malignant HCC phenotypes.

A number of chronic liver pathologies including fibrosis and cirrhosis are strongly associated with the development of HCC (32). One in three patients with cirrhosis will develop HCC in their life (33). Dysregulation of the TGF-*β*/SMAD family was confirmed in the setting of liver fibrosis and cirrhosis (34). Subsequent evidence demonstrated that miRNAs were involved in both liver fibrosis and cirrhosis *via* direct targeting of SMAD proteins (35). The promotion of epithelial–mesenchymal transition and cancer cell metastasis by TGF-*β* and the SMAD family was reported in a great number of malignancies. Of note, SMAD7 is known to be one of the most significant regulators of the TGF-*β* pathway. Our findings highlighted the role of SMAD7 in HCC pathogenesis. The absence of SMAD7 was widely noted in HCC tissues compared to background liver tissue, and a pronounced decline in SMAD7 levels in rat models with advanced liver fibrosis and cirrhosis was found as compared to normal rat liver tissue. In addition, SMAD7 deletion was consistently noted in HCC cell lines (**Figure S2B**). We confirmed SMAD7 to be a direct target of miR-21-3p in HCC. Restoration of SMAD7 levels was found to impair Huh-7 and Hep-G2 cell migration and invasion capabilities, which were noted to be enhanced by miR-21-3p. GSVA analysis revealed SMAD7 to be mainly involved in the TGF-*β* signaling transduction pathway in HCC. Deletion of SMAD7 in the progression of HCC is thus of vital importance.

Previous genetic analysis of miR-21-3p targets confirmed that miR-21-3p is strongly associated with the Hippo signaling pathway. Cross-talk between the TGF-β/SMAD and Hippo/YAP1 signaling pathways has also been widely studied  (36, 37). YAP1, a protein that interacts with SMAD7, plays diverse roles in various illnesses (22). Increased levels of SMAD7 were observed in approximately 50% of pancreatic cancer cases, with increased SMAD7 and YAP1 mRNA expression found to contribute to resistance against TGF-*β* signaling in this condition (38). Intranuclear SMAD7 promotes YAP1 translocation from the nucleus to the cytoplasm and blocks YAP1 transcription, inhibiting HCC progression (39). In the cytoplasm, YAP1 facilitates SMAD7 to activated T*β*RI and inhibits the TGF-*β*/SMAD signal transduction. The counterbalance between SMAD7 and YAP1 exerts a great impact on the TGF-*β* signal transduction. Here, we explored interactions among miR-21-3p, SMAD7 and YAP1. Our findings revealed YAP1 expression to be increased in HCC tissues compared to background liver tissue. In the advanced stages of liver disease in rat models, YAP1 expression was higher than in normal liver tissue. Overexpression of miR-21-3p was found to enhance YAP1 expression; YAP1 overexpression *via* miR-21-3p was partly reversed by SMAD7 transfection. Increased levels of YAP1 were found to promote the expression of nuclear transcription factor CTGF. Lower SMAD7 expression facilitates the intranuclear entry of YAP1 and the subsequent initiation of downstream factor transcription. *In vitro* experiments revealed this phenomenon to be more pronounced in Huh-7 as compared to Hep-G2 cells, and this is likely of relevance to intracellular miR-21-3p and SMAD7 expression [**Figure 1A(c)**, **S2B**].

**Figure 7:**
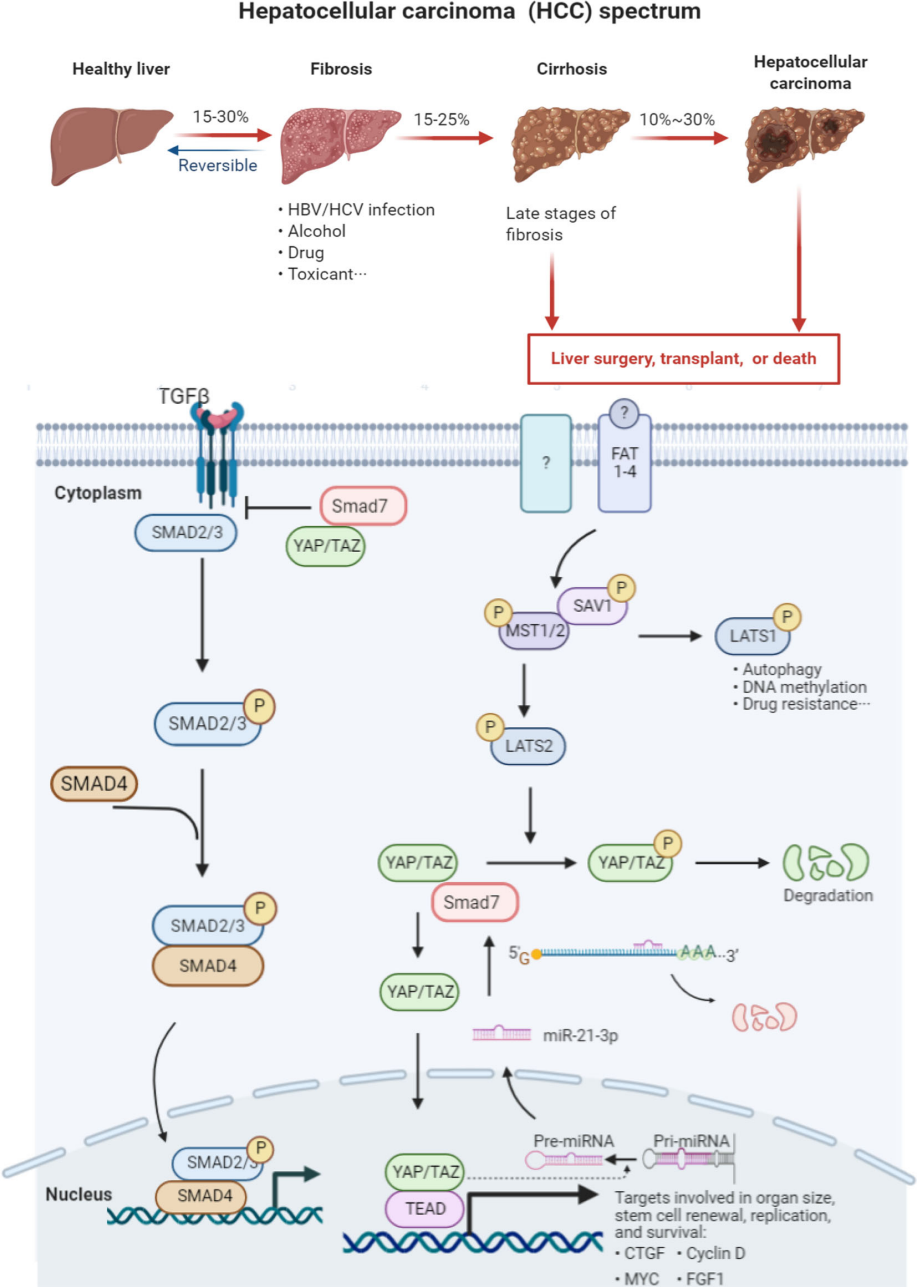
Graph abstract HCC manifestation is typically preceded by hepatic fibrosis and cirrhosis. There are limited cures for patients suffering advanced liver cirrhosis and HCC. Dysfunction of the Hippo signaling and TGF-beta transduction pathways plays a vital role in the pathogenesis and course of this illness. YAP1 recruits SMAD7 to activated TGF-*β* receptor I (T*β*RI), hampering the formation of co-SMAD (SMAD2/3 and SMAD2/4) complexes. These SMAD complexes are thus unable to enter the nucleus to activate transcription of downstream effectors. SMAD7 serves as the negative regulator of the TGF-β signaling pathway. The Hippo signaling pathway is composed of a series of kinases including MST1/2, LATS1/2, and nuclear effector YAP1. Once the Hippo pathway is activated, LATS1/2 is phosphorylated by MST1/2. LATS1 is mainly involved in cell autophagy, DNA methylation and drug resistance. LATS2 phosphorylates YAP1, leading to its degradation. Overexpressed miR-21-3p directly silences SMAD7 expression by binding to its 3′-UTR region, further impairing the linkage between SMAD7 and YAP1. The decreased stability of the SMAD7/YAP1 complex facilitates YAP1 translocation to the nucleus and results in the subsequent transcription of the downstream gene connective tissue growth factor (CTGF).

Recent studies have suggested that not only is YAP1 regulated by a series of miRNAs, but that nuclear YAP1/TAZ (the paralog of YAP1) influences the conversion of pri-miRNA into pre-miRNA (40). The Hippo/YAP1 signaling pathway is currently understood to be the key pathway that converts physicomechanical into biochemical signals to affect cellular functions. The characteristic microenvironment that distinguishes HCC from other malignancies is an increase in local mechanical stress and stiffness. Over half of HCC manifests in the setting of existing cirrhosis. Hepatic fibrosis and cirrhosis are typical examples of extracellular matrix sclerosis (41). Although data were varied among studied patients, the stiffness of HCC tissue (55 kpa) was reported to be almost 10 times that of normal liver tissue (4 kpa) as detected by Fibroscan (42). Previous studies confirmed YAP1 to be a mechano-transducing effector that translocates from the cytoplasm to the nucleus when cells are shifted from soft to stiff matrices (43–45), consistent with our findings studying rat model fibrosis and cirrhosis. We supposed that throughout the pathogenesis of HCC, not only does stiffness increase but the increased synthesis of YAP1 *via* miR-21-3p likely results in nuclear translocation and further facilitation of pre-miR-21-3p formation (**Figure 7**). However, such possible positive feedback and the cascade amplification response brought about in HCC require further cell fractionation and *in vivo* confirmatory experiments.

In conclusion, our results underscored the oncogenic role of miR-21-3p in HCC. MiR-21-3p-SMAD7/YAP1 axis was identified to better understand the mechanisms of occurrence and development of liver cancer.

## Data Availability Statement

The datasets presented in this study can be found in online repositories. The names of the repository/repositories and accession number(s) can be found in the article/**Supplementary Material**.

## Ethics Statement

The studies involving human participants were reviewed and approved by the local Research Ethics Committee at Zhongnan Hospital of Wuhan University (Approval No.2018078). The patients/participants provided their written informed consent to participate in this study. The animal study was reviewed and approved by the Animal Care and Use Committee of Wuhan University following the Animal Experiment/Animal Biosafety Level-III Laboratory Guidelines.

## Author Contributions

Methodology: YH, MY, and FW. Experiments: YH Bioinformatic analysis: YH, MY, and FW. Grammar Correction: CW, JieL, and JiaL. Sample collection: YH, CW, and JieL. Supervision: JF, JingL, QZ, LL, and YC. Funding: QZ and YC. All authors contributed to the article and approved the submitted version.

## Funding

This work was supported by research grants from the National Natural Science Foundation of China (No. 81670554); the Science and Technology Plan of Wuhan City (2020020601012208); the Natural Science Fund for Distinguished Young Scholars of Hubei Province (No. 2017CFA068) and the National Key R&D Program of China (No. 2019YFC0121505).

## Conflict of Interest

The authors declare that the research was conducted in the absence of any commercial or financial relationships that could be construed as a potential conflict of interest.
