# Supplementary figures and images for: MiR-21-3p Promotes Hepatocellular Carcinoma Progression via SMAD7/YAP1 Regulation
Source: Front Oncol. 2021 Mar 8;11:642030. doi: 10.3389/fonc.2021.642030 (PMC7982593; doi:10.3389/fonc.2021.642030)

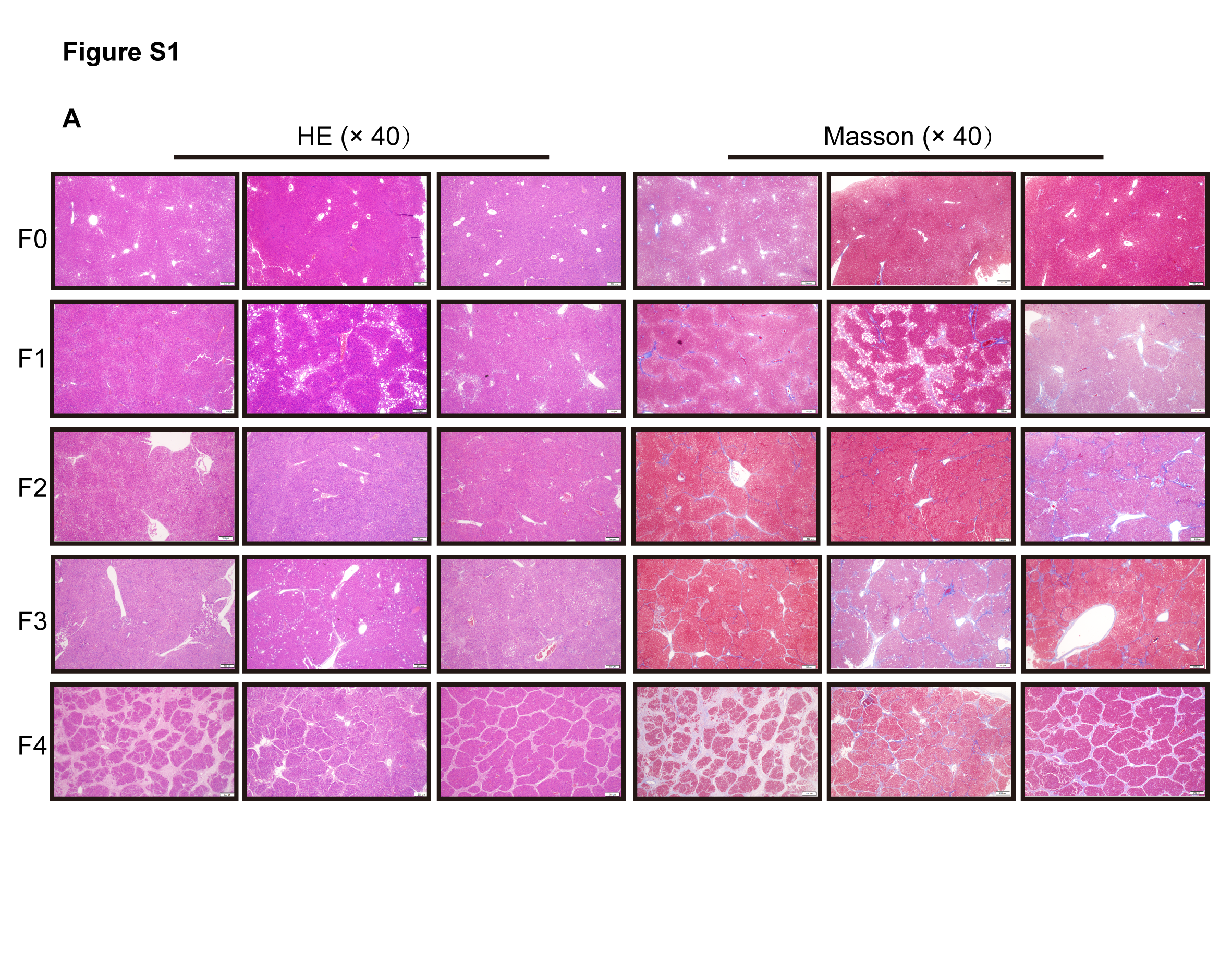

Supplement: Supplementary file 1 [file Image_1.tif]

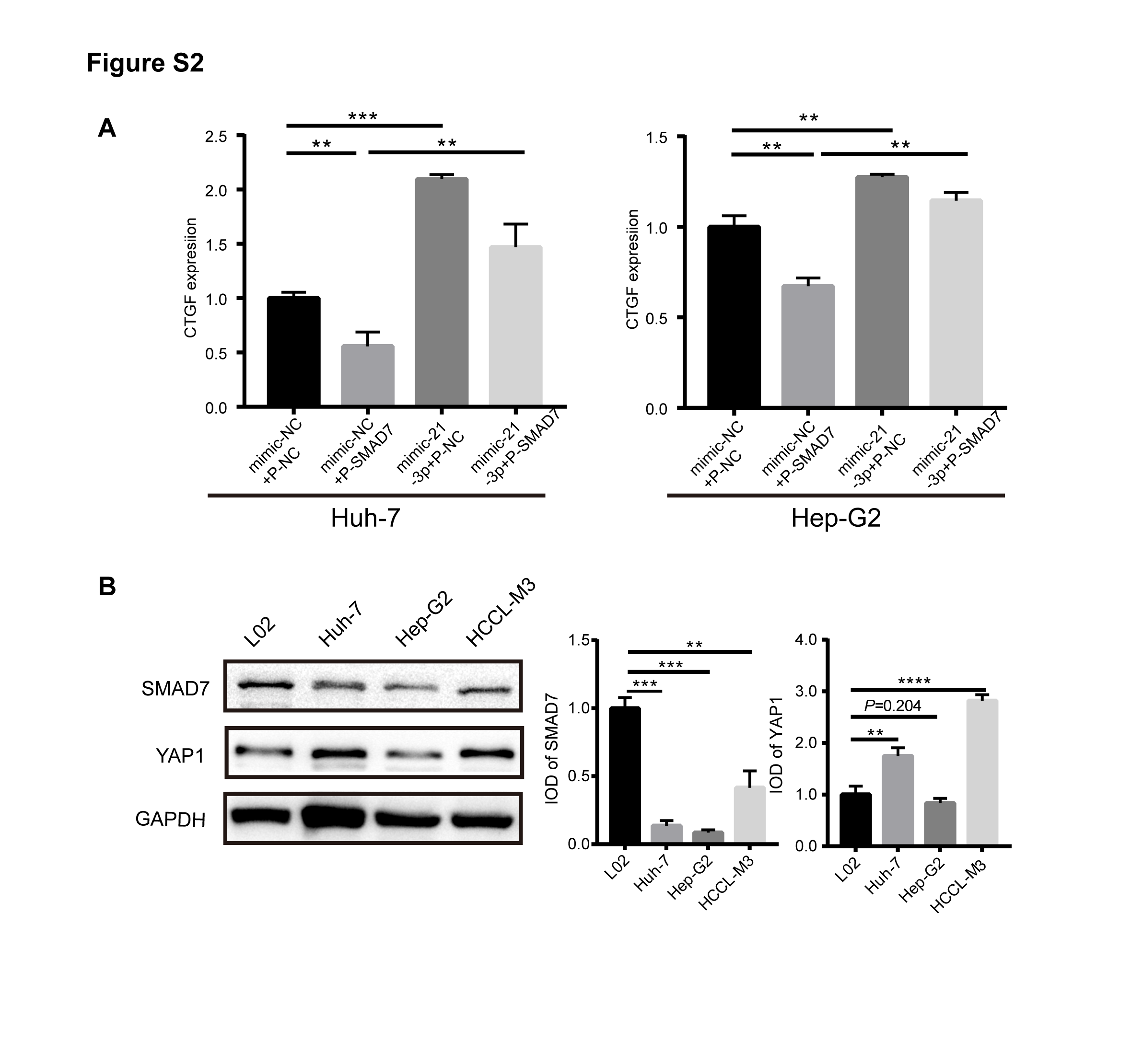

Supplement: Supplementary file 2 [file Image_2.tif]
