# Supplementary material for: MiR-21-3p Promotes Hepatocellular Carcinoma Progression via SMAD7/YAP1 Regulation
Source: Front Oncol. 2021 Mar 8;11:642030. doi: 10.3389/fonc.2021.642030 (PMC7982593; doi:10.3389/fonc.2021.642030)
Supplement: Supplementary file 3 [file Table_1.docx]

Table S1 Gene primers for qRT-PCR in this article.

| Genes | Sense (5’-3’) | | Antisense (5’-3’) |
| --- | --- | --- | --- |
| SMAD7 | | TTCAGGACCAAACGATCTGCG | GATGGTGGTGACCTTTGGCAC |
| YAP1 | | GAACTCGGCTTCAGGTCCTC | GGTTCATGGCAAAACGAGGG |
| LATS2 | | TGGAATGCCAACAATGTAGCG | ATTATCACTCTCTCCAGGGGCG |
| CTGF | | AGAGGGCTGTCGGCG | CACAGGAGCTGGTGTTCCAT |
| GAPDH | | AGAAGGCTGGGGCTCATTTG | GCAGGAGGCATTGCTGATGAT |
